# Design of inductive electrostatic boom spray system based on embedded closed electrode structure and droplet distribution test in soybean field

**DOI:** 10.3389/fpls.2024.1367781

**Published:** 2024-06-17

**Authors:** Changxi Liu, Jun Hu, Rui Cao, Yufei Li, Shengxue Zhao, Qingda Li, Wei Zhang

**Affiliations:** ^1^ College of Engineering, Heilongjiang Bayi Agricultural University, Daqing, China; ^2^ Heilongjiang Province Conservation Tillage Engineering Technology Research Center, Daqing, China; ^3^ Key Laboratory of Soybean Mechanized Production, Ministry of Agriculture and Rural Affairs, Daqing, China; ^4^ Beidahuang Group Heilongjiang Nenjiang Farm Co., Ltd., Heihe, China

**Keywords:** induction electrostatic spray, boom sprayer, electrode, droplet deposition, soybean spraying

## Abstract

The large water demand, insufficient deposition on the back of the leaf and the uneven distribution of droplets are the problems of traditional agricultural ground plant protection machinery, which leads to low agricultural control efficiency. Combined with the advantages of electrostatic spray technology and the characteristics of high working efficiency and low probability of droplets drift of ground sprayer, an inductive electrostatic boom spray system based on embedded electrode structure is designed and mounted on a large self-propelled boom sprayer for field testing. Based on the working characteristics of the fan nozzle and the analysis of the theory of charge, the inductive electrostatic spray device is designed. The performance of the device is tested and the rationality of the system design is verified by COMSOL numerical simulations, charge-to-mass ratio, and particle size distribution measurements. The spray deposition scanning software and the Box-Behnken experimental design method are used to analyze the spray droplet deposition rate and coverage density of the sprayer on the front and back of the target leaves. The results show that the embedded closed electrode structure designed in this paper can avoid the problem of electrode wetting, and the electric field generated by it is mainly concentrated in the spray liquid film area, and the intensity reaches 6~7 V/m. At the conventional application height (500 mm), the maximum charge-to-mass ratio is 2.91 mC/kg, and the average particle size is 168.22 μm, which is 12.87% lower than that of ordinary spray, when the spray pressure is 0.3 MPa and the electrostatic voltage is 12 kV. The results of field experiments show that the optimum combination of the working parameters with the spray speed is 8.40 m/s, the spray pressure is 0.35 MPa, the charging voltage is 11.50 kV, the amount of droplet deposition in the lower dorsal area of the blade is 1.44 µL·cm^-2^. This study can provide a certain basis for the application of electrostatic spray technology in ground sprayers.

## 1 Introduction

Plant protection spraying in field management can effectively reduce the impact of pests and diseases, and ensure the yield and quality of crops (He, 2019; Liu et al., 2023a). However, due to the relative backwardness of China’s plant protection pesticide application technology and equipment, the ground sprayer has problems such as large water demand, insufficient deposition on the back of the leaf and uneven distribution of fog droplets during plant protection operations, resulting in a large number of pesticide residues and pollution of agricultural products, soil and water bodies (Chambers et al., 2014). Due to the different planting patterns, planting crops and topography in various regions, there are many types of plant protection machinery, mainly including knapsack manual (motorized) sprayer, self-propelled boom sprayer, towed (suspended) boom sprayer, aviation (manned, unmanned) plant protection aircraft (He, 2017). At present, China is still dominated by man-knapsack and small power plant protection equipment, which has many problems such as high labor intensity, poor uniformity and low efficiency (Liu et al., 2021). Aviation plant protection operation has the advantages of high operation efficiency, not limited by field terrain, strong adaptability and good protection for plant protection personnel, but the high maintenance cost, poor endurance and safety problems of this plant protection operation mode restrict its rapid development (Huang, 2011; Cunha et al., 2017). Compared with other plant protection methods, the large self-propelled boom sprayer has the advantages of high work efficiency, less missing spraying and great pesticide distribution uniformity. However, the existing self-propelled boom sprayer still stays at the level of large-capacity and large-droplet spraying, and there are problems of large water demand and insufficient amount of sedimentation on the back of the blade during operation. Therefore, the development of new and efficient plant protection equipment and the improvement of pesticide spraying technology are the research directions of plant protection experts at home and abroad. Among them, electrostatic spray technology is a new type of pesticide application technology developed on the basis of droplet control technology and the theory and practice of ultra-low volume spray (Patel et al., 2015; Lin et al., 2023). Compared with conventional spray technology, electrostatic spray can use the electrostatic surround effect to reduce pesticide drift, environmental pollution, improve pesticide utilization rate, and save pesticide application costs. Law and Lane et al. have done a lot of research on the application of electrostatic spray technology to crops and agricultural products (Law and Lane, 1981) and Sinha, Pascuzzi Matthews, kabashima, Palumbo, Coates, Bowen, and Sumner et al. have successfully applied electrostatic spray technology to greenhouses and orchards (Pascuzzi and Cerruto, 2015; Sinha et al., 2020). However, most of China’s research on electrostatic spray devices is still in the theoretical and laboratory research stage, and only some of them have been carried on drones for relevant experimental research. According to the characteristics of a variety of agricultural aviation aircraft, Ru et al. (Ru et al., 2011) developed an electrostatic spray system and a high-voltage power supply system, and installed the designed double-nozzle aviation electrostatic sprinkler on the Y5B fixed-wing aircraft for field insect control experiments, and the results showed that the insect control effect was increased by nearly 50%. Jin et al. (Jin and Ru, 2016) optimized the aviation electrostatic sprinkler and preliminarily studied and designed an unmanned helicopter aviation electrostatic spray system based on the research foundation of their team in recent years. The test results show that under the action of electrostatic electricity, the atomization effect of the droplets is better, and the adhesion of the droplets is stronger.

After years of basic theoretical research by most scholars in China, electrostatic spray technology has gradually transitioned from laboratory theory to scientific and technological products and field devices (Maski and Durairaj, 2010; He, 2022). However, most of the electrostatic nozzles used by the existing electrostatic spray technology on the boom sprayer are derived from the knapsack electrostatic sprayer or the aviation electrostatic sprayer, and the interaction with the boom sprayer is poor, and it is difficult to install and disassemble (Zhao et al., 2024). In addition, the electrostatic spray device with bare electrodes currently used has the problem of plate wetting after long-term use, resulting in a short circuit in the electrostatic system and a decrease in charge performance. The research of Patel (Patel et al., 2017) and Lan et al. (Lan et al., 2018) also showed that different electrode materials have different charging effects on the droplets, and the droplets are adsorbed in reverse on the electrode, which is easy to wet the induction electrode, resulting in leakage or unchargeability, and affecting the charging stability of the electrostatic spray system.

Based on the above research background, an inductive electrostatic spray system suitable for the use of boom sprayers is designed, and the electrostatic spray device used with an embedded closed electrode structure can effectively solve the problem of unchargeable electrode caused by wet plates. The large electrostatic boom sprayer equipped with an electrostatic spray system shows good droplet coverage and adhesion to the back of the leaf in the soybean field droplet deposition test. This study provides a certain design basis for the future production of integrated electrostatic spray system, and also provides a reference for the promotion of electrostatic spray technology for anti-drift spray operations.

## 2 Materials and methods

### 2.1 Design of an electrostatic spray device embedded in a closed electrode

**Figure 1:**
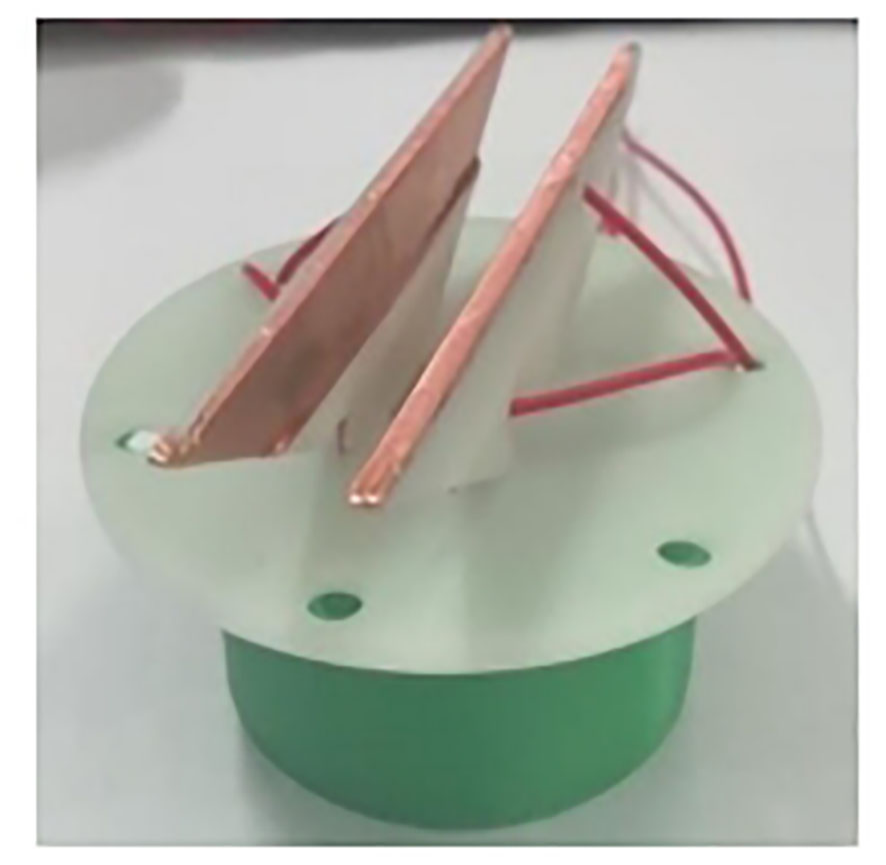
Electrode bare electrostatic spray device.

The electrostatic nozzle is the implementation component of the inductive electrostatic spray charge, which plays a key role in the electrostatic spray effect. At present, the induction electrodes of most electrostatic spray devices are exposed (**Figure 1**), which makes the induction electrodes extremely wet due to the effect of electrostatic adsorption.

And after a long time of contact with air and water, the electrode is easy to corrode, rust and even lose its electrostatic effect (**Supplementary Figure S1**).

In this paper, an inductive electrostatic spray device based on an embedded closed electrode structure was designed. It can be divided into electrode layer and spray water film layer along the direction of the nozzle, and the electrode plate, parallel plate surface and air layer can be directly regarded as parallel electrode dielectric capacitors (Li et al., 2019; Liu et al., 2023b. The amount of charge on the capacitor plate is shown in Equation 1:


(1) $$C=\frac{\varepsilon_{r}S}{4\pi kd}$$

(2) $$Q=CU=\frac{U\varepsilon_{r}S}{4\pi kd}$$

Where: *ε_r_* is dielectric constant of air, *S* is plate area, *k* is electrostatic force constant, *d* is the plate distance, *U* is charge voltage.

As can be seen from Equation 2, the value of *Q* increases as *U* and *S*. Assuming that the medium and voltage are constant, the charge is related to the distance *d* between the two plates. Due to the sputtering phenomenon of the liquid during spraying, if the distance d between the plates is too small, the droplets may be deposited on the surface of the electrode, resulting in tip discharge. Assuming that the distance between the electrode and the liquid film is *d*/2 and *d*=2*j*+*M*, the dielectric constant of this composite medium *ε_z_* is equivalent as shown in Equation 3 (Song et al., 2017):


(3) $$\varepsilon_{z}=\frac{\varepsilon_{j}\varepsilon_{r}d}{2(J+\varepsilon_{j}M)}$$

Where: *J* is thickness of the electrostatic cover, *M* is thickness of air layer, *ε_J_* is relative permittivity of the electrostatic cover.

The charge *Q* is as shown in Equation 4 (Patel et al., 2016):


(4) $$Q=\frac{U\varepsilon_{j}\varepsilon_{r}S}{8\pi k(J+\varepsilon_{j}M)(2J+d)}$$

**Figure 2:**
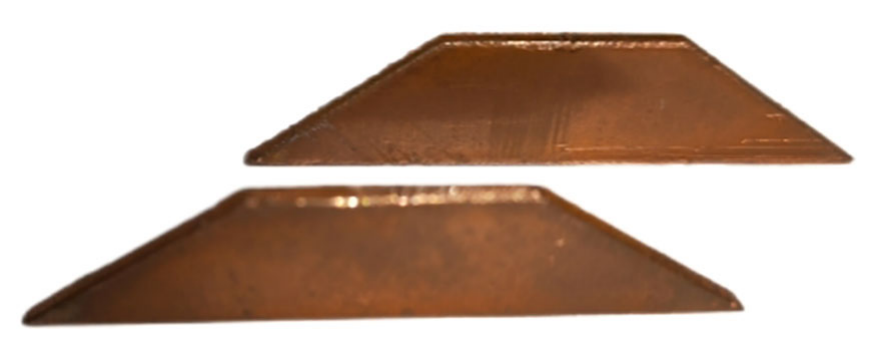
Embedded electrode plates.

In order to increase the charge of the spray more effectively, according to the working characteristics and charge theory analysis of the fan nozzle commonly used in the boom sprayer, the electrode form was designed as a parallel induction electrode parallel to the fan-shaped spray. The electrode consists of two parallel trapezoidal copper plates in the shape of two trapezoidal plates with an upper 20 mm, a lower 52 mm, a height of 15 mm, and a thickness of 2 mm (**Figure 2**).

The electrostatic nozzle designed in this study is divided into two parts, the upper part is the connection area and the lower part is the working area.

**Figure 3:**
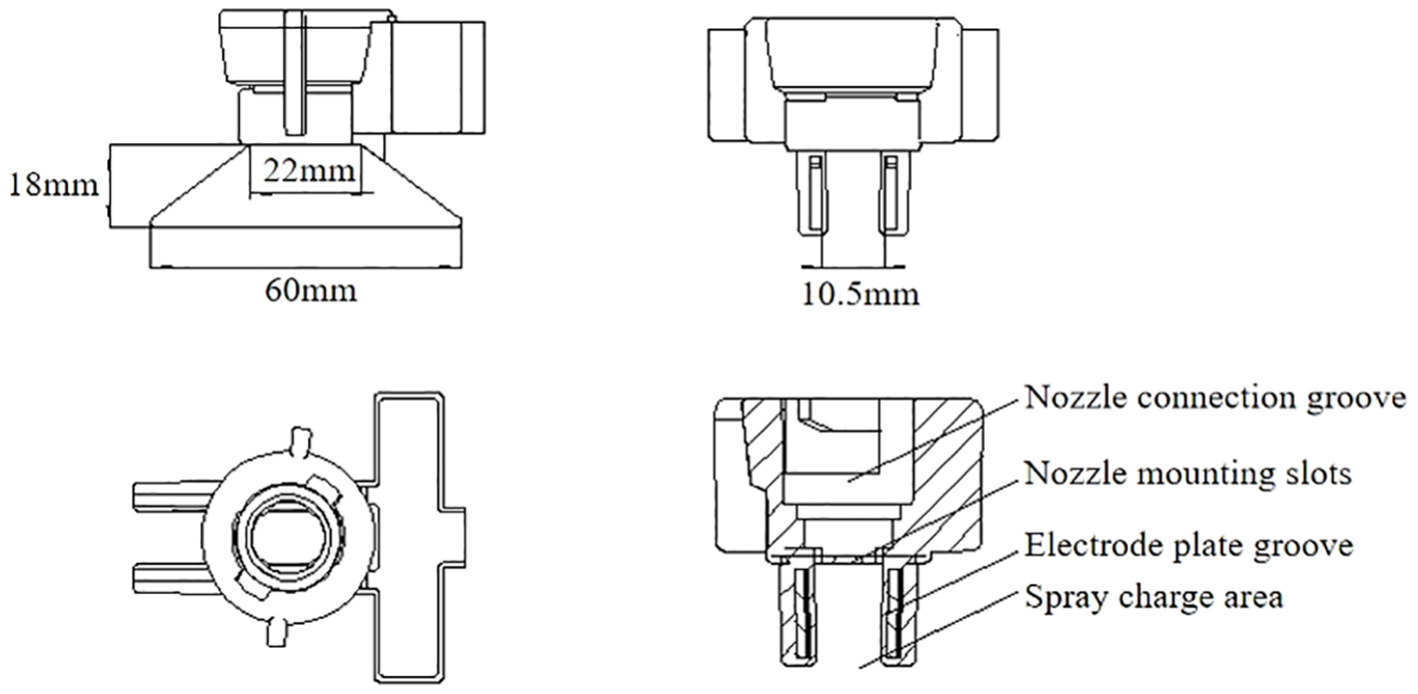
Structure and size of electrode plate embedded induction electrostatic spray device.

**Figure 4:**
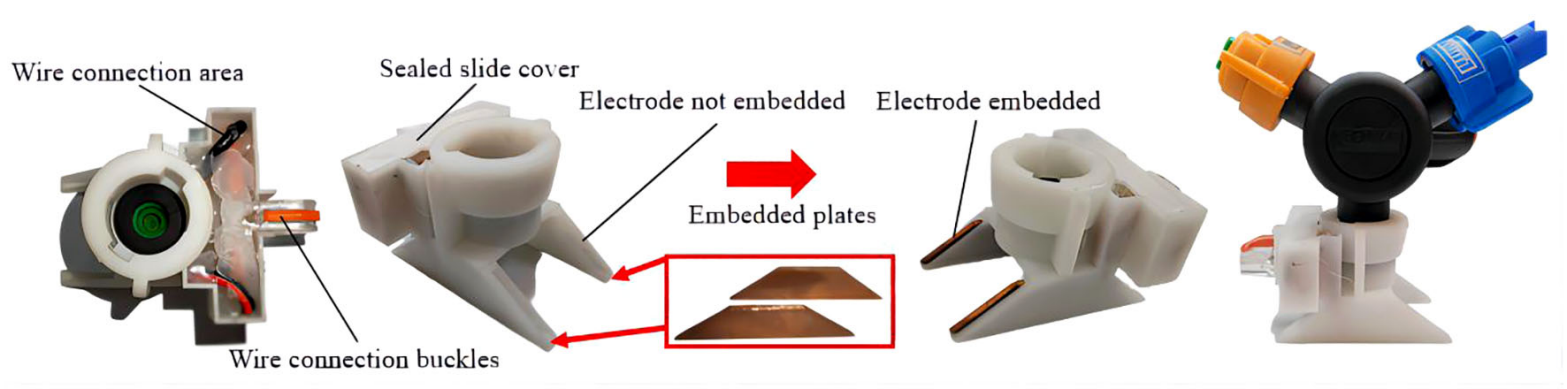
Inductive electrostatic spray device with electrode plate embedded.

As shown in **Figure 3**, the upper part of the device adopts a rotating buckle design to connect with the nozzle, and the internal structure of the nozzle cap is convenient for installing the fan nozzle at the connection of the device. The rubber gasket mouth can be installed in the nozzle connection groove, and the wine connection area is in the right groove. The electrode plate groove is the installation area of two parallel trapezoidal electrode plates, and the plates are all wrapped in insulating materials to avoid the phenomenon of fog droplet adsorption and wetting after long-term power supply. In addition, the closed plate placement slows down corrosion, extends service life, and provides a more stable electrostatic field. As shown in **Figure 4**, the design of the electrostatic spray device is similar to that of the traditional nozzle nozzle cap, which makes the electrostatic spray device more versatile and can be used with a variety of fan-shaped nozzles. The innovative design of the induction electrostatic spray unit is 3D printed and modeled in epoxy resin.

### 2.2 Space electric field simulation of inductive electrostatic spray device

In order to verify that the inductive electrostatic spray device designed in this paper still has a good space electric field sensing effect when the insulating material is encapsulated, the COMSOL Multiphysics 5.6 numerical simulation software is used to simulate the space electric field (Almamury, 2015).

#### 2.2.1 Numerical simulation methods

The model wizard under initialization conditions is selected as a spatial dimension 3D, and the physics is set to an AC/DC electrostatic field. And the steady-state model is selected in the overall preset study and the software initialization is completed. The UG 10.0 3D model of the induction electrostatic spray device was imported. As shown in **Supplementary Figure S2**, the material definition and simulation process of the built model are demonstrated.

#### 2.2.2 Parameter setting and meshing

**Figure 5:**
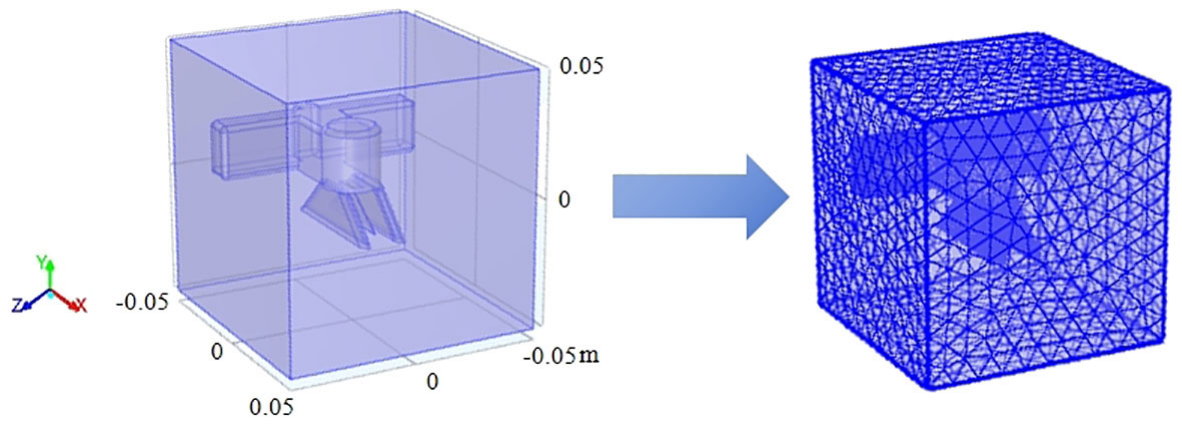
Model import and meshing.

The electrostatic nozzle and nozzle housing material is set to insulating epoxy resin and set to zero voltage. Since the liquid film flows out through the nozzle body, it is set to the ground value. In order to evaluate the optimal charge and space electric field on the surface of the liquid film, a voltage of 14 kV is loaded onto the induction electrode plate according to previous laboratory studies. After evaluating all the conditions, the structural mesh is given by the software, as shown in **Figure 5**.

### 2.3 Design of an induction electrostatic boom spray system

The power supply, the relay, the electrostatic generating part and the electrostatic nozzle form the inductive electrostatic spray system. During operation, the power is transmitted to the relay component by the power supply component, and the power supply mode is converted into an intermittent pulse form in the relay, which is amplified by the high-voltage electrostatic and transmitted to the electrostatic nozzle. Compared with other plant protection equipment, the long working width and the large number of nozzles are the uniqueness of the boom sprayer. In this paper, according to the characteristics of the boom sprayer, a circuit connection distribution diagram of an electrostatic spray device is designed, as shown in **Supplementary Figure S3**.

### 2.4 Selection of power supply and electrostatic generator

According to the Technical Requirements for Electrostatic Sprayers (GB/T33006–2016) and combined with theoretical calculations, the JDFS-01 adjustable electrostatic generator of Shenzhen-Hong Kong Electronics Co., Ltd. was selected, and the parameters are shown in **Supplementary Table S1**.

**Figure 6:**
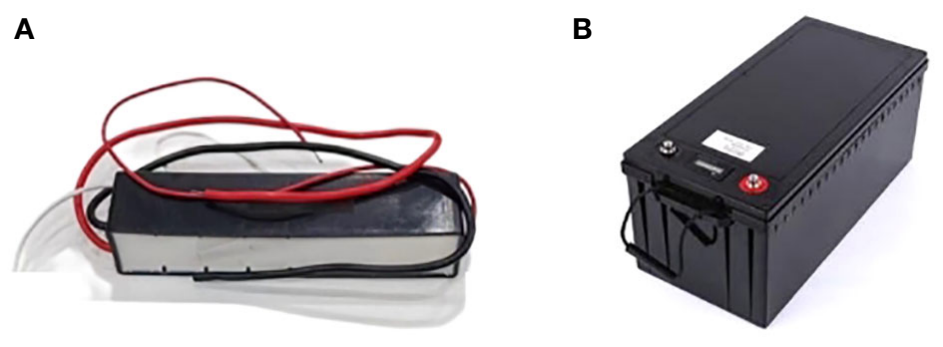
Selection of electrostatic generators and lithium battery. **(A)** Electrostatic generator, **(B)** Lithium battery.

The spray width is 32 m, and the number of nozzles is 64, which is the configuration of a large boom sprayer. Using the JDFS-01 electrostatic generator selected above, the power supply of the electrostatic spray device of the boom sprayer requires at least 160 Ah of electrical energy. Through theoretical calculations and multi-faceted reference and selection, the 200 Ah capacity 12 V lithium battery produced by Jiangsu New Energy Electrical Appliance Manufacturing Co., Ltd. is selected as the power source of the electrostatic spray system. The electrostatic generator and lithium battery used in this paper are shown in **Figure 6**.

### 2.5 Measurement of droplet size and charge-to-mass ratio

The Winner 318 laser particle size meter is used for the particle size test of charged droplets, and its measurement accuracy was 0.001 μm (Jinan Micro Nano Technology Co., Ltd. Shandong, China). The charging voltage of the droplet size test is set at 0∼12 kV and the test position is 500 mm below the nozzle. The spray pressure is set to 0.3 MPa, and each test is repeated 3 times, and the results are averaged. The droplet charge-to-mass ratio measurement is carried out using the Faraday cylinder charge-to-mass ratio test device of the Key Laboratory of Jiangsu University. The ammeter in this device is a Model 6485 picoammeter (measurement accuracy ± 0.5%), and the electronic balance is a JA31002 precision electronic balance (measurement accuracy 0.001 g). In order to collect accurate and stable current values, the droplet charge-to-mass ratio test is set to a charging voltage of 6∼12 kV, and the time of each spray is 60 s. The spray pressure is set to 0.3 MPa, and each test is repeated 3 times, and the results are averaged. SPSS 19.0 is used for multivariate ANOVA.

### 2.6 Soybean field droplet deposition characteristics test using an inductive electrostatic spray device

#### 2.6.1 Test equipment and location

**Figure 7:**
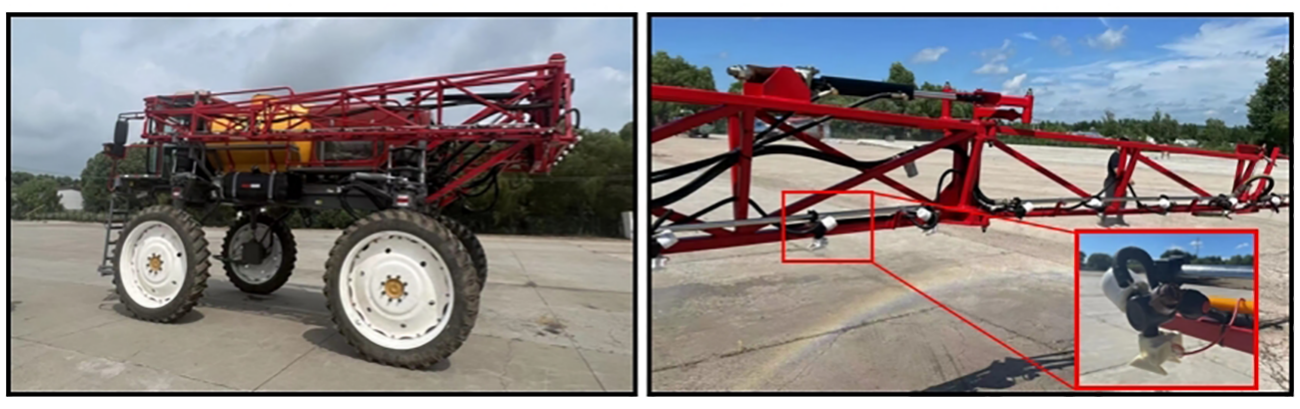
Induction electrostatic spray system installation site.

According to the requirements of GB/T17997–2008 “Field Operation Procedures and Spraying Quality Assessment of Pesticide Sprayers (Devices)”, spray tests were conducted from July 2 to July 14, 2022 in No. 6 standardized farmland in the first management area of Heshan Farm, Jiusan Management Bureau, Nenjiang City, Heilongjiang Province. The induction electrostatic spraying device designed in this experiment is equipped with a boom sprayer as shown in **Figure 7**, and the sprayer model is the 3WX-3000 self-propelled boom sprayer developed by the China Academy of Agricultural Mechanization, and its machine parameters are shown in **Supplementary Table S2**.

#### 2.6.2 Sampling point layout and test method

**Figure 8:**
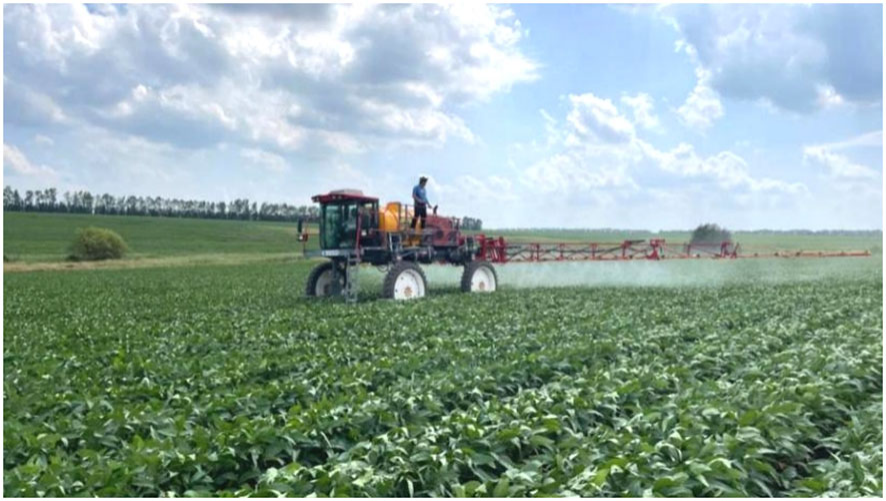
Electrostatic spraying operation site in soybean field.

The center line of the boom sprayer is the axis of both sides of the left and right, 2 m away from the central axis is set up as the first group of test points, 4 m away from the right (left) side of the central axis is set up as the second group of test points, 8 m away from the right (left) side of the central axis is set up as the third group of test points, and 2 m away from the rear of each test point is set up the second row of test points. There are two rows of test points, each row of 6 test points, a total of 12 test points. The site of electrostatic spraying in the soybean field is shown in **Figure 8**. The arrangement of water-sensitive paper is shown in **Supplementary Figure S6**.

**Figure 9:**
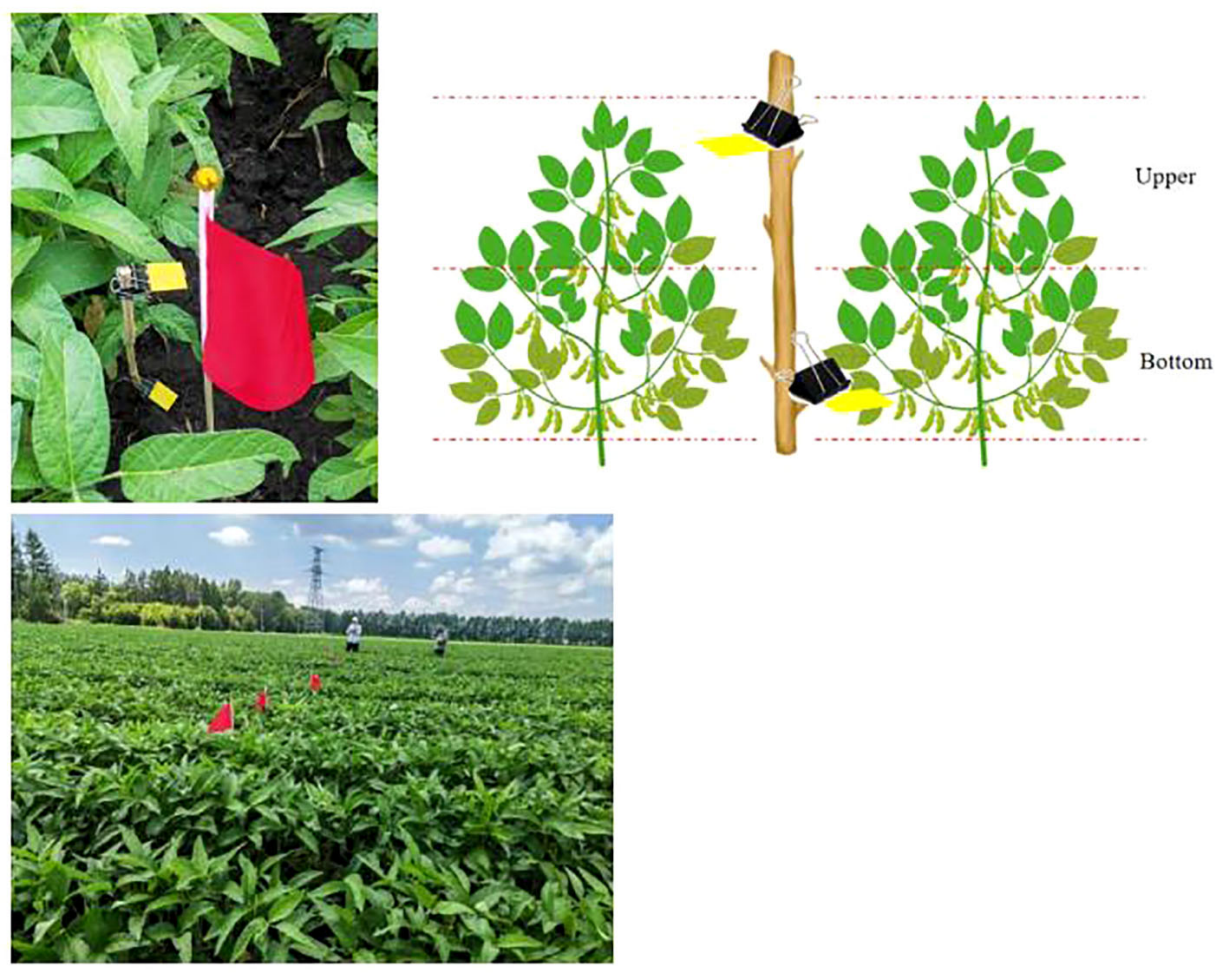
Diagram of the layout of water-sensitive paper.

25×25 water-sensitive paper (Syngenta Crop Protection GmbH) is used to analyze the distribution of droplets generated under each test combination. During the application period, the growth cycle of soybean (variety is Longken 3092) is about 65 days, and it is in full flowering stage at the time of application, and the height is about 50 cm. As shown in **Figure 9**, two sheets of water-sensitive paper are arranged in the upper and lower areas of each soybean plant, and a total of four water-sensitive papers are used for one target. It is fixed to the simulated target using metal clips and randomly placed in the soybean canopy. In order to facilitate the subsequent collection of water-sensitive paper, flagpoles are placed on each simulated target for marking.

After spraying, the water-sensitive test strips are collected and stored in a ziplock bag, and the droplets are analyzed by the Deposit scan software after the amount of deposition are analyzed. **Supplementary Figure S4** depicts the procedure for droplet size analysis using stereo micro scope with Deposit scan software. The image processing procedure used in the study was similar to what Martin (Martin, 2014) reported previously. DepositScan software for finding droplet deposition rate (µL·cm^−2^) as per the work of Zhu (Zhu et al., 2011).

## 3 Results

### 3.1 Simulation analysis of space electric field of induction electrostatic spray device

**Figure 10:**
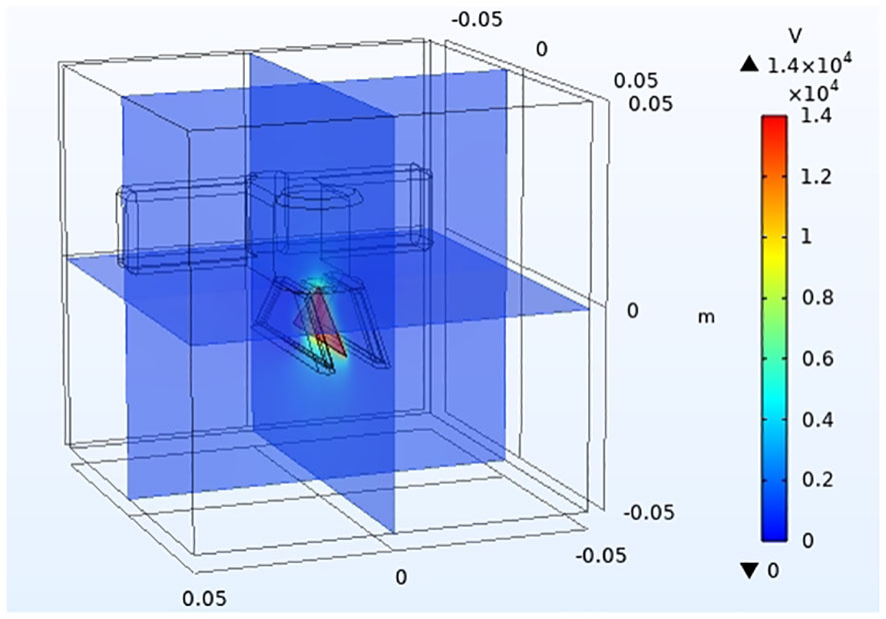
Potential distribution of electrostatic spray device in three-dimensional space.

**Figure 11:**
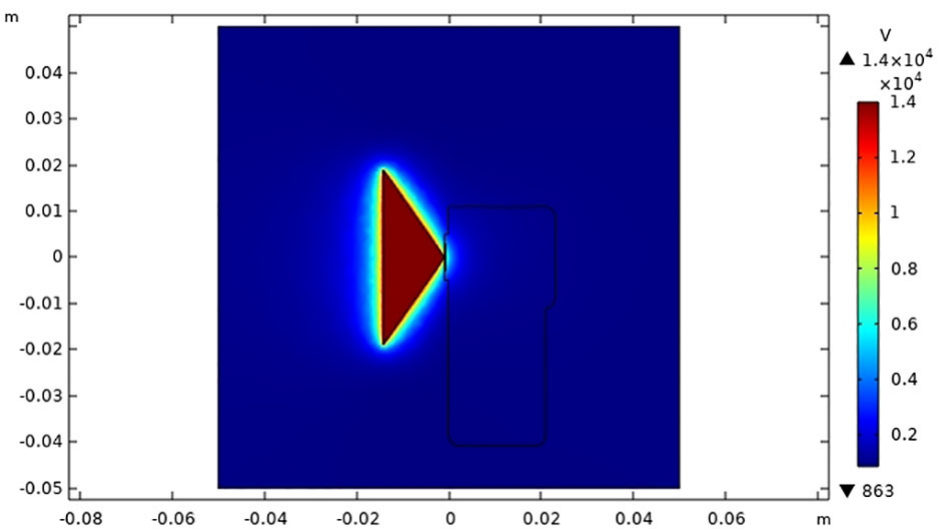
Potential distribution of the two-dimensional in-plane electrostatic spray device section.

As shown in **Figures 10**, **11**, the space electric field simulation effect of the induction electrostatic spray device shows good results. The liquid film of the fan-shaped nozzle is in the induced electric field, and the overall charge uniform electric potential on its surface reaches 1.4×10^4^ V/m. The surface potential of the pulse induction electrostatic nozzle is extremely low, which indicates that the way in which the pulse induction electrostatic nozzle is wrapped with insulating material is theoretically correct, and can give sufficient charge performance to the spray fan without affecting the charging effect.

It can be seen in the overall space electric field **Supplementary Figure S5** that the electric field is mainly concentrated in the strongest area of the spray liquid film domain, and the electric field intensity reaches 6∼7×10^4^ V/m. The electric field intensity gradually decays from the liquid film area to both sides, and the electric field intensity of part of the pulse-induction electrostatic sprinkler decreases to less than 3∼4×10^4^ V/m. The impact of static electricity on the boom sprayer and other electrical components is small, which reduces the impact of electrostatic soft breakdown, and ensures the operation performance of the electrostatic spray and boom sprayer.

### 3.2 Measurement results and analysis of charge-to-mass ratio and particle size of electrostatic droplets

As shown in **Table 1**, the electrostatic spray charge-to-mass ratio measurements are given at different charging voltages and different spray heights.

**Table 1:** Values of charge-to-mass ratio.

Spray height (mm) Charging voltage (kV)	6	8	10	12
Charge-to-mass ratio (mC/kg)				
200	1.83	2.56	2.94	2.96
500	1.73	2.40	2.87	2.91
800	1.61	2.21	2.78	2.82

SPSS is used to perform a multivariate ANOVA for electrostatic spray charge-to-mass ratios at different voltages and distances. The charge-to-mass ratio is designed as the dependent variable, and the charging voltage and spray distance are designed as the independent variables. The effect of the significant difference between charging voltage and spray distance on charge-to-mass ratio was analyzed.

As shown in **Table 2**, it is concluded that the minimum load-to-mass ratio is 1.61 mC/kg under the condition that the spray distance is 70 cm and the voltage is 6 kV. Under the condition that the spray distance is 200 mm and the voltage is 12 kV, the maximum load-to-mass ratio is 2.96 mC/kg, which exceeds the national standard of 0.8 mC/kg for electrostatic spray. Compared with the bare electrode electrostatic spraying device developed by our team, its charge-to-mass ratio has increased by 0.5 mC/kg under the same spraying conditions (Liu et al., 2023b). As can be seen from **Table 2**, the effects of voltage and distance on the charge-to-mass ratio are statistically significant. Both the charging voltage and the distance can affect the charge-to-mass ratio, but the charging voltage has a greater effect. This is because the charging voltage is the main factor determining the charge-to-mass ratio, and the upward trend of the voltage in the range of 6 kV to 10 kV is significantly larger, and the increase of the voltage-to-load-mass ratio after 10 kV is significantly smaller.

**Table 2:** Subjective effect test between voltage, spray distance and charge-to-mass ratio.

Test of subjective effects						
Dependent variable: charge-to-mass ratio (mC/kg)						
	Sum of squares	df	Mean square	F	Sig.	Partial eta side
Type	2.797^a^	5	0.559	248.937	0.000	0.995
Calibrate model	73.112	1	73.112	32534.403	0.000	1.000
Intercept	2.702	3	0.901	400.786	0.000	0.995
Voltage (kV)	0.095	2	0.048	21.163	0.002	0.876
Distance (cm)	0.013	6	0.002			
Error	75.923	12				
Total	2.811	11				
Total of the corrections	73.112	1	73.112	32534.403	0.000	1.000

^a^ *R^2^* = 0.995 (Adjusted *R^2^* = 0.991).

### 3.3 Electrostatic droplet size measurement results and analysis

**Figure 12:**
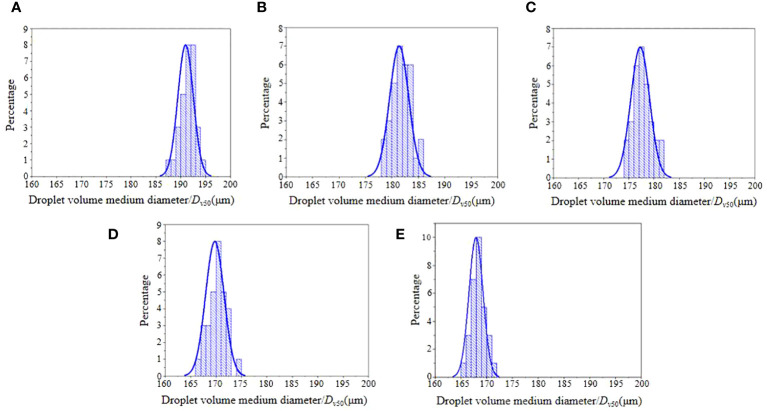
Particle size distribution of electrostatic droplets. **(A)** Voltage of 0kv, **(B)** Voltage of 6kv, **(C)** Voltage of 8kv, **(D)** Voltage of 10kv, **(E)** Voltage of 12kv.

As shown in **Figure 12**, the nozzle is measured three times at the electrostatic voltage of 0 kV, 6 kV, 8 kV, 10 kV, and 12 kV, and the particle size data of the droplet *D_v_*_50_ is obtained.

As shown in **Table 3**, the SPSS multiple comparative analysis method was used to evaluate the droplet *D_v_*_50_ particle size data for electrostatic spray and conventional spray at different voltages.

**Table 3:** Table of results of multiple comparison of droplet size.

Multiple comparison results						
Dependent variable: Droplet *D_v_*_50_ particle size (μm)						
	(I) Voltage (kV)	(J) Voltage (kV)	Mean Difference (I-J)	Standard error	Confidence interval	
					Lower limit	Upper limit
LSD	0.00	6.00	10.42*	0.35	9.73	11.11
		8.00	14.82*	0.35	14.13	15.51
		10.00	21.54*	0.35	20.85	22.23
		12.00	24.66*	0.35	23.97	25.35
	6.00	0.00	-10.42*	0.35	-11.11	-9.73
		8.00	4.40*	0.35	3.71	5.09
		10.00	11.12*	0.35	10.43	11.81
		12.00	14.24*	0.35	13.55	14.93
	8.00	0.00	-14.82*	0.35	-15.51	-14.13
		6.00	-4.40*	0.35	-5.09	-3.71
		10.00	6.72*	0.35	6.03	7.41
		12.00	9.84*	0.35	9.15	10.53
	10.00	0.00	-21.54*	0.35	-22.23	-20.85
		6.00	-11.12*	0.35	-11.81	-10.43
		8.00	-6.72*	0.35	-7.41	-6.03

*Extremely significant.

As the electrostatic voltage increased, the particle size of *D_v_*_50_ decreased by 5.44%, 7.73%, 11.23%, and 12.87%, respectively, when compared to conventional spray at 6∼12 kV electrostatic spray.

The particle size of droplet *D_v_*_50_ decreases significantly, when the charging voltage increases from 0 to 10 kV. When the electrostatic voltage exceeds 10 kV, the decreasing trend slows down. According to the theoretical analysis, it can be seen that the degree of ionization of electrons inside the droplet increases when the electrostatic voltage increases. When the outward force of the electrons breaks through the surface tension of the liquid medicine, the droplets are further broken. Since the number of electrons that the droplets can carry is limited, if the voltage continues to increase when the electron separation effect is maximum, the force generated by the electrons will not be enough to break through the surface tension limit of the droplet, so the droplets cannot be further refined.

### 3.4 Results and analysis of droplet deposition characteristics in soybean field

**Figure 13:**
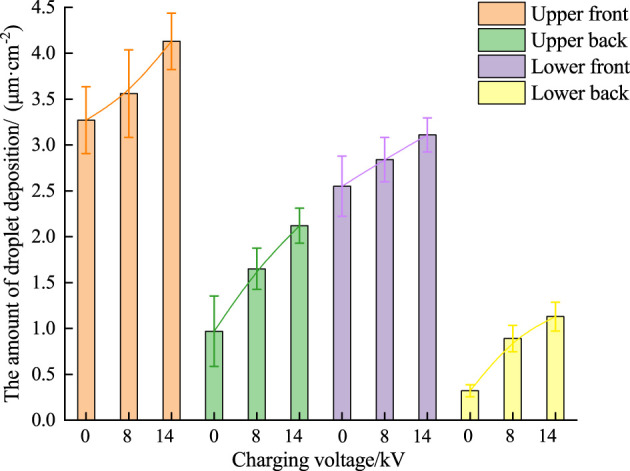
Amount of droplet deposition in different collection areas.

**Figure 14:**
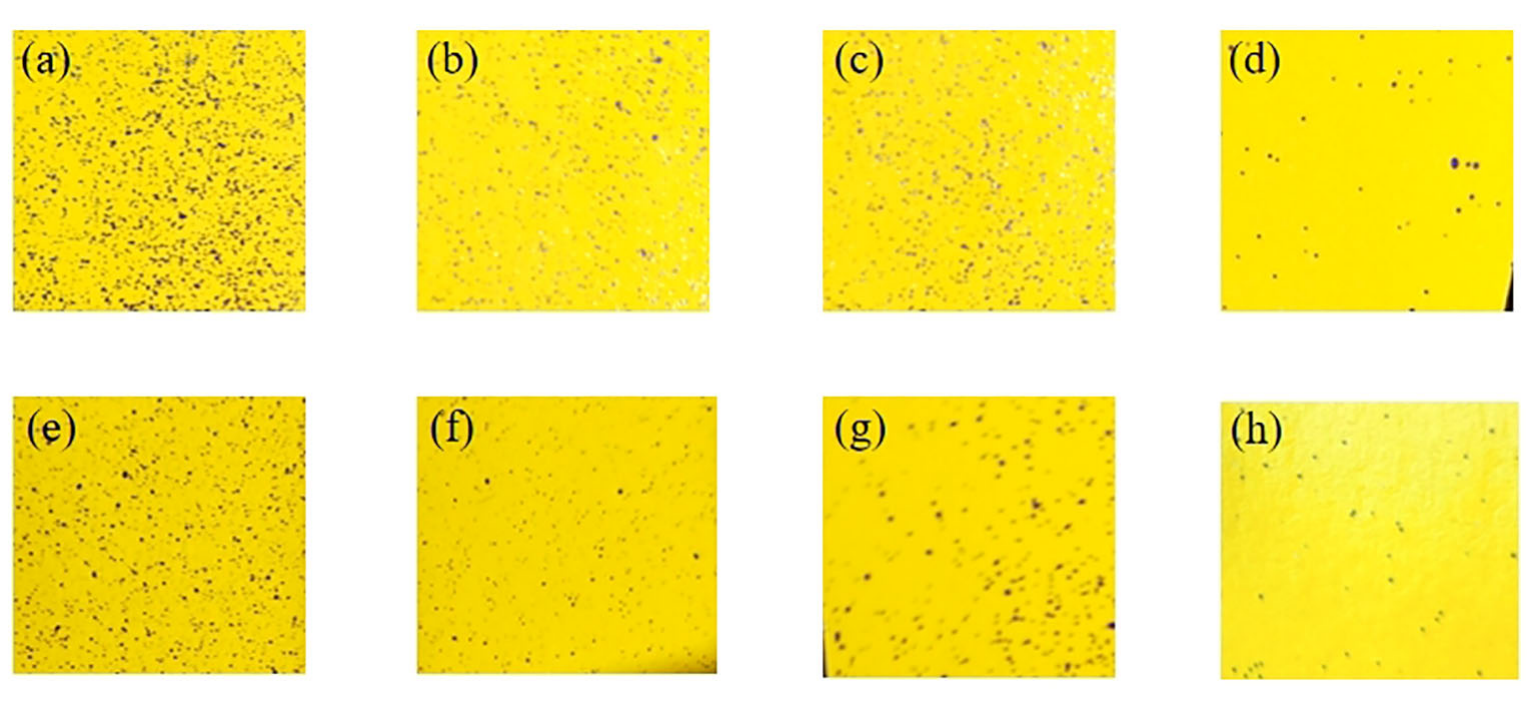
The water-sensitive paper collects the results of the amount of droplet deposition in different areas. **(A)** Upper front with electricity, **(B)** Upper front without electricity, **(C)** Lower front with electricity, **(D)** Lower front without electricity, **(E)** Upper back with electricity, **(F)** Upper back without electricity, **(G)** Lowerback with electricity, **(H)** Lower back without electricity.

The sampling locations were divided into upper and bottom layers. The average droplet deposition of each sample obtained by DepositScan software is presented in **Figure 13**. The attachment of the water-sensitive paper is shown in **Figure 14**.

It is observed from **Figure 13** that the droplet deposition amount decreased from the upper to the lower layer, and droplet deposition amount in the upper (3.27 ± 0.36 and 0.97 ± 0.12 µL·cm^−2^), and lower layers (2.55 ± 0.32 and 0.32 ± 0.06 µL·cm^−2^) are found in locations when no static electricity is applied. The amount of droplet deposition increased with the increase of charging voltage, and the amount of deposition in the upper part of the leaf is the largest, and the amount of deposition on the back of the lower part of the leaf is the smallest. Compared with conventional spraying, a 3 fold increase in droplet deposition in the lower back part of the leaf can be observed with a 14 kV charging voltage. Similarly, a 1.5 fold increase in droplet deposition is observed in the upper back part of the leaf. The rest of the front area also showed an increase in the amount of droplet deposition. The findings of this research are in line with previous research and hence confirms that the electrostatic spray technology can significantly improve the adhesion ability of droplets in the ventral shelter of the plant (Ru et al., 2011; Cunha et al., 2017).

### 3.5 Multivariate orthogonal test based on Box-Behnkenof droplet deposition in soybean field

In order to further explore the optimal field spraying operation parameters of the electrostatic spraying device designed in this paper, Box-Behnken is used as an experimental design method. The paper sets out a series of experiments on the spray speed (the levelvalue X_1_, the coded value x_1_), the spray pressure (the level-value X_2_, the coded value x_2_), the charging voltage (the level-value X_3_, the coded value x_3_). The multi-factor orthogonal test is conducted to obtain the optimal deposition amount of droplets on the back of the lower layer, and the optimal operating parameters are explored. The level-value and the coded value of the experiment elements are shown in **Table 4**. The experimental scheme and the result are shown in **Table 5**.

**Table 4:** Experiment factor level and coded value.

Coded value	The spray speed/(m·s^-1^)	The spray pressure/(MPa)	The charging voltage/(kV)
-1	6	0.2	8
0	8	0.3	11
1	10	0.4	14

**Table 5:** The experimental scheme and the result.

No.	The spray speed, x_1_	The spray pressure, x_2_	The charging voltage, x_3_	Droplet deposition,Y
1	-1	-1	0	0.61
2	1	-1	0	0.42
3	-1	1	0	1.31
4	1	1	0	0.88
5	-1	0	-1	1.04
6	1	0	-1	0.68
7	-1	0	1	1.39
8	1	0	1	1.01
9	0	-1	-1	1.01
10	0	1	-1	1.03
11	0	-1	1	0.94
12	0	1	1	0.87
13	0	0	0	1.24
14	0	0	0	1.25
15	0	0	0	1.23
16	0	0	0	1.12
17	0	0	0	1.17

This research imports the experimental data in Design-Expert 10.0 to make a regression fit, which sets up the regression model of the droplet deposition evaluation score from different elements, as shown in Equation 5.

After getting rid of the non-distinctive regression items, the regression model of the droplet deposition is shown in Equation 6:


(5) $$\begin{array}{l} Y=1.44-0.11X_{1}+0.14X_{2}+0.11X_{3}-0.14X_{1}X_{2}+0.052X_{1}X_{3}\\ -0.19X_{2}X_{1}-0.036X_{1}^{2}+0.22X_{2}^{2}-0.031X_{3}^{2} \end{array}$$

(6) $$Y=1.44-0.11X_{1}+0.14X_{2}+0.11X_{3}-0.14X_{1}X_{2}-0.19X_{2}X_{1}+0.22X_{2}^{2}$$

**Figure 15:**
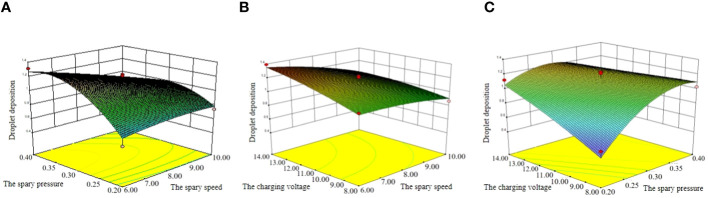
Response surfaces of interactive factors influence on test indexes. **(A)** Y=f(x_1_, 0, x_3_), **(B)** Y=f(0, x_2_, x_3_), **(C)** Y=f(x_1_, x_2_, 0).

In the regression equation, one element with the factor level 0 is randomly selected, and the remaining two elements are studied to find out their influence on amount of droplet deposition. The software Design-Expert 10.0 is used to make an analysis to get the response hook face affected by the interaction factors, as shown in **Figure 15**.

In **Figure 15A**, if the charging voltage is fixed, the droplet deposition increases first and then decreases with the increase of spray pressure. With the increase of the spray speed, the amount of droplet deposition is more suitable in the range of spray speed 7~9 m/s and spray pressure 0.25~0.35 MPa.

In **Figure 15B**, if the spray pressure is fixed, the amount of droplet deposition increases relatively steady with the increase of charging voltage. With the increase of spray speed, the amount of droplet deposition is more suitable in the range of charging voltage 9~13 kV and spray speed 7~9 m/s.

In **Figure 15C**, this paper finds that if the spray speed is fixed, the amount of droplet deposition increases with the increase of charging voltage and spray pressure. The amount of droplet deposition is more suitable in the range of charging voltage 9~13 kV and spray pressure 0.25~0.35 MPa. In this case, the contour lines of the response surface are closed ellipses, indicating that the interaction between conical wind speed and spray pressure is strong and has a maximum value. The above analysis is consistent with the significance shown in Equation 6.

Based on the working performance demand and the actual working condition of the induction electrostatic spray device, this work plans to succeed in the lower charging voltage, the higher amount of droplet deposition. According to the different elements having different effects, this paper needs to optimize all results. This paper regards amount of droplet deposition as an objective function, makes the optimization design to 3 experimental elements, including the harging voltage, the spray height and the spray pressure. The optimization constraint conditions can be conducted as follows:


(7) $$\{\begin{array}{c} \max Y(\text{μL}\cdot {\;}^{\text{cm}}{\;}^{-2})\\ s.t.\{\begin{array}{c} X_{1}\in (7.00\text{m/s},9.00\text{m/s})\\ X_{2}\in (0.25\text{MPa},0.35\text{MPa})\\ X_{3}\in (9.00\text{kV},13.00\text{kV}) \end{array} \end{array}$$

Where: *X*_1_ is spray speed, *X*_2_ is spray pressure, *X*_3_ is charging voltage.

The influence laws of three experimental factors affecting the amount of droplet deposition is comprehensively considered to get the best parameter combination, using Design-Expert 10.0 software to make an optimization solution. This research gets the optimum working parameter combination, the spray speed being 8.42 m/s, the spray pressure being 0.32 MPa, the charging voltage being 11.55 kV, the amount of droplet deposition being 1.46 µL·cm^-2^.

In order to use the optimum parameter combination in the actual spray operation, this paper makes the round number of them, the spray speed is 8.40 m/s, the spray pressure is 0.35 MPa, the charging voltage is 11.50 kV, 3 repetitive tests are made to get the average value, the amount of droplet deposition is 1.44 µL·cm^-2^ which means the experimental results keep an accord with the theoretical results substantially, thus the regression model is great.

## 4 Discussion

During the use of induction electrostatic spray device, the researchers mainly studied the relationship between operation parameters and spraying quality. As researchers believe that the atomization effect of aviation electrostatic spray device plays a major role in pest control (Pascuzzi and Cerruto, 2015; Jin and Ru, 2016), the device is often directly used for testing, while ignoring the uniqueness of field boom sprayer compared with aviation aircraft in most field crop pesticide application studies. Besides, different electrode materials have different charging effects on the droplets, and the droplets are adsorbed in reverse on the electrode, which is easy to wet the bare induction electrode, resulting in leakage or unchargeability, and affecting the charging stability of the electrostatic spray system. In order to improve the phenomenon of electrode wetting, an embedded closed electrode structure is innovatively designed, and the distribution of the induced electric field is described by numerical simulation. The results clearly show that the embedded electrode structure can solve the problem of electrode wetting without affecting the charging effect.

The charge-to-mass ratio is the key parameter of the design of induction electrostatic spray device since the higher charge-to-mass ratio, a stronger electrostatic surround phenomenon can be generated, which in turn causes more droplets to be adsorbed (Maski and Durairaj, 2010; Patel et al., 2017; Lan et al., 2018). However, the effect of multifactorial spray deposition, including the charge-to-mass ratio, should be of more concern (Cunha et al., 2017; Huang, 2011). These studies have shown that identifying the influencing factors of electrostatic spray device and stabilizing ascent based on operating conditions is a necessary scientific issue. On the basis of previous research results, the results of the multivariate interaction of the deposition of fog droplets in the belly of the soybean canopy are given, including the charging voltage. It is found that the spray pressure is still the most important factor affecting the amount of droplet deposition, but the right charging voltage can improve the detrimental effects of spray speed. With the increase of charging voltage, the growth trend of droplet coverage gradually slows down. Therefore, mastering the charging characteristics and the influence law of droplet deposition can achieve the ideal spray effect.

## 5 Conclusions

In order to solve the problem of wet electrode plates in the exposed electrodes, an induction electrostatic spraying system suitable for field boom sprayers was designed by numerical simulation and multi-factor field experiments. The research conclusions are as follows:

(1) The embedded closed electrode structure designed in this paper can well meet the charging requirements and solve the problem of electrode wetness. The electric field generated by it is mainly concentrated in the spray liquid film area, and the intensity reaches 6∼7 V/m. The maximum charge-to-mass ratio of the electrostatic spray device is 2.91 mC/kg, and the average particle size is 168.22 μm, which is 12.87% lower than that of ordinary spray.
(2) Compared with conventional spraying, a 3 fold increase in droplet deposition in the lower back part of the leaf can be observed with a 14 kV charging voltage. Similarly, a 1.5 fold increase in droplet deposition is observed in the upper back part of the leaf.
(3) The Box-Behnken was introduced to design the experimental methods, making the series experiments on the operation parameter of the induction electrostatic spray device, finding out that the optimum working parameter combination is the spray speed is 8.40 m/s, the spray pressure is 0.35 MPa, the charging voltage is 11.50 kV, and the amount of droplet deposition being 1.44 µL·cm^-2^.

## Data availability statement

The original contributions presented in the study are included in the article/**Supplementary Material**. Further inquiries can be directed to the corresponding author.

## Author contributions

CL: Conceptualization, Data curation, Formal analysis, Visualization, Writing – original draft, Writing – review & editing. JH: Conceptualization, Data curation, Formal analysis, Funding acquisition, Writing – original draft, Writing – review & editing. RC: Conceptualization, Data curation, Supervision, Writing – original draft. YL: Data curation, Formal analysis, Methodology, Supervision, Writing – review & editing. SZ: Formal analysis, Methodology, Resources, Validation, Writing – original draft. QL: Data curation, Formal analysis, Investigation, Visualization, Writing – review & editing. WZ: Formal analysis, Funding acquisition, Project administration, Writing – original draft.
