# Supplementary material for: Design of inductive electrostatic boom spray system based on embedded closed electrode structure and droplet distribution test in soybean field
Source: Front Plant Sci. 2024 Jun 17;15:1367781. doi: 10.3389/fpls.2024.1367781 (PMC11215122; doi:10.3389/fpls.2024.1367781)
Supplement: Supplementary file 1 [file DataSheet_1.docx]

**Table S1.** Parameters of electrostatic generator

| Product name | Electrostatic generator | Product model | JDFS-01 |
| --- | --- | --- | --- |
| Product size | 120*28*25 mm | Shell mechanism | Square one-piece |
| Working voltage | 12 V | Working current | 1A |
| Output voltage | 5⁓20 kV | Output current | 150 mA |
| Power | 2 W | Continuous working hours | 24 h |
| Input line length | 26 cm | Output line length | 27 cm |
| Arc length | / | Weight | 154 g |
| Product feature：Continuous power-on no-load | | | |

**Table S2.** Main technical parameters of boom spray

| Types | Parameters |
| --- | --- |
| Overall dimensions (L*W*H)（mm×mm×mm） | 8500×2800×3900 |
| Output power/kW | 162 |
| Structural mass/t | 8.5  （without liquid medicine） |
| Wheel track/mm | 2800⁓3800 |
| Wheel base/mm | 3800 |
| Minimum ground clearance of chassis/mm | 1750 |
| Ground speed/（km/h） | 32 |
| Container capacity/L | 2030 |
| Work breadth/mm | 2400 |
| Height of the spray rod from the ground/mm | 480⁓3000 |
| Nozzle spacing/mm | 500 |
| Number of nozzle/n | 48 |

**
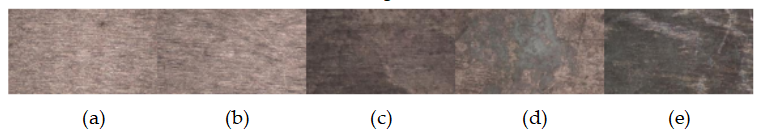
**

Figure S1. Electrode corrosion degree appearance. (a) Not corrosion, (b) Mild corrosion, (c) Moderate corrosion, (d) Strong corrosion, (e) Severe corrosion

**
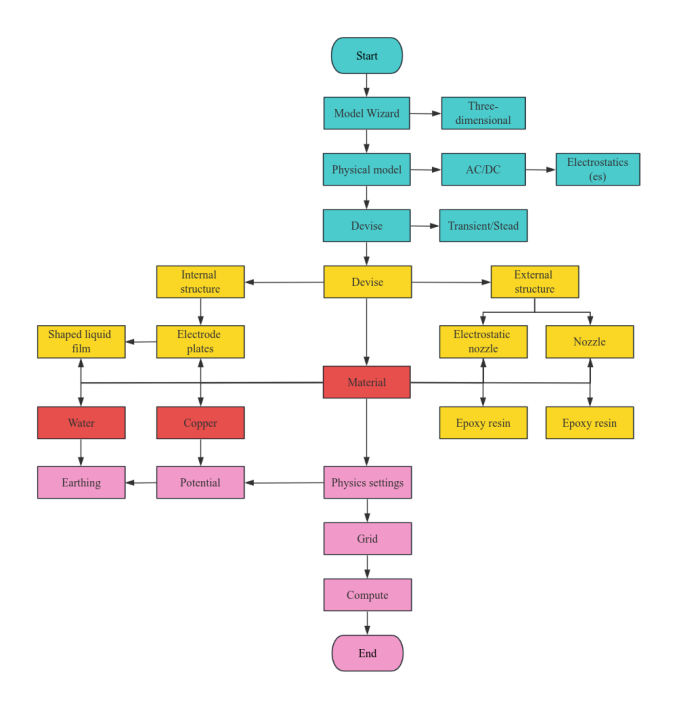
**

Figure S2. Inductive electrostatic spray device with electrode plate embedded

**
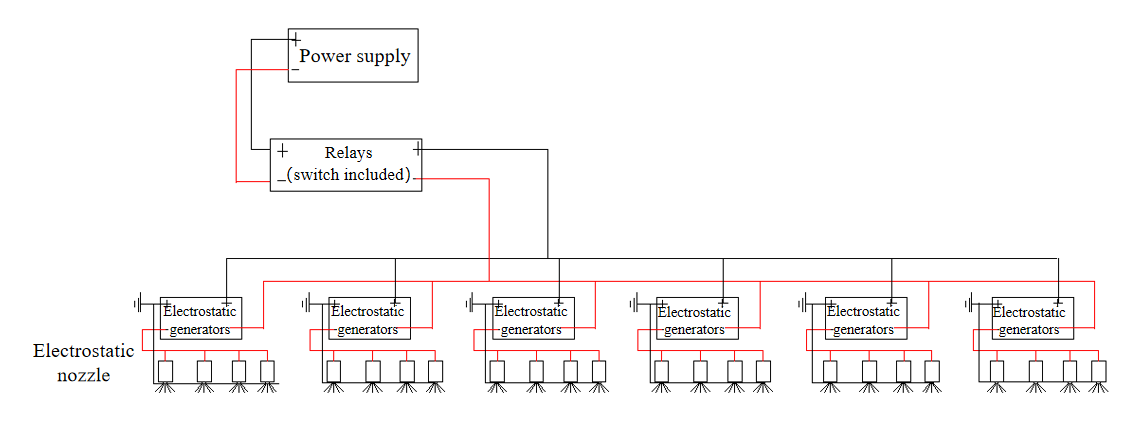
**

Figure S3. Circuit connection diagram of induction electrostatic spray device

**
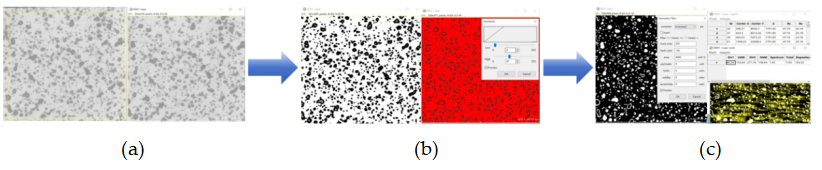
**

Figure S4. Deposit scan software was used to analyze the coverage and deposition density of droplets on water-sensitive paper. (a) Area selection, (b) Setting the scale (left) and adjusting the contrast threshold (right), (c) Fragmentation (left) and result display (right)

**
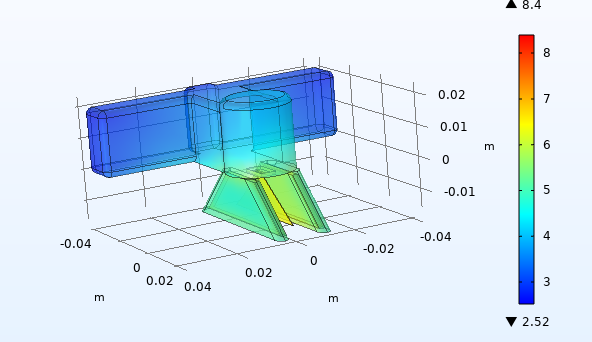
**

Figure S5. Electric field distribution of the electrostatic spray device in three-dimensional space


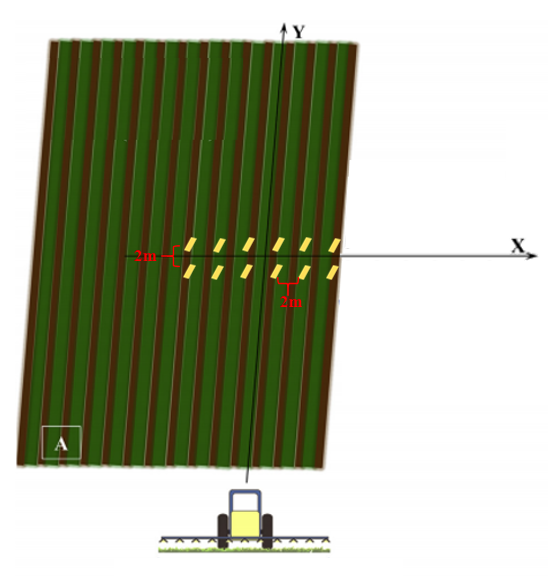


Figure S6. Schematic diagram of electrostatic spraying operation in a soybean field
